# The ICS triad in critical illness: a time-dependent pathological driver of mortality and organ dysfunction

**DOI:** 10.3389/fmed.2026.1833525

**Published:** 2026-07-14

**Authors:** Valery V. Likhvantsev, Levan B. Berikashvili, Mikhail Ya. Yadgarov, Alexey A. Yakovlev, Andrey V. Grechko

**Affiliations:** Federal Research and Clinical Centre of Intensive Care Medicine and Rehabilitology, Moscow, Russia

**Keywords:** chronic critical illness, immunosuppression and catabolism syndrome, persistent inflammation, PICS, RICD

## Abstract

**Background:**

Advances in intensive care have increased acute-phase survival, leading to a growing population of patients with prolonged ICU stays, a condition termed chronic critical illness (CCI). A key pathophysiological candidate is the triad of persistent inflammation, immunosuppression, and catabolism (ICS), yet its relationship to CCI and its independent prognostic impact remain poorly defined. The objective of this study was to investigate the impact of developing the ICS on hospitalization outcomes in critically ill patients.

**Materials and methods:**

We conducted a real-world data analysis of adult ICU patients using electronic health records from RICD (December 2017–July 2023). ICS was defined by concurrent C-reactive protein >20 mg/L, albumin <30 g/L, and lymphocytes <0.8 × 10^9/L. Outcomes included ICS incidence, mortality, organ support requirements, and multiple organ failure. Statistical analyses involved Kaplan–Meier estimates, Cox regression, and multivariate modeling.

**Results:**

Among 1,963 analyzed patients, 540 (27.5%) developed ICS. The cumulative risk of ICS was 21.3% by day 28 and 76.1% by week 16 of ICU stay. Patients with ICS had significantly higher hospital mortality (31% vs. 4.9%, *p* < 0.001), greater need for mechanical ventilation (67% vs. 41%, *p* < 0.001) and vasopressors (33% vs. 8.6%, *p* < 0.001). In multivariable analysis, ICS was an independent risk factor for hospital mortality (HR = 2.16, 95% CI 1.28–3.65, *p* = 0.004) and for multiple organ failure (HR = 1.25, 95% CI 1.05–1.47, *p* = 0.011).

**Conclusion:**

The ICS triad is a common, time-dependent syndrome that independently predicts adverse outcomes, including hospital mortality and organ failure, in critically ill patients. Our data support the notion that ICS may play a role in the pathological substrate of CCI, and suggest the existence of a potentially modifiable ‘prolonged critical state’ that could precede the development of CCI. These findings highlight the potential value of shifting some clinical focus toward earlier recognition and prevention of the ICS triad, alongside the management of established CCI.

## 1 Introduction

Progress in intensive care has led to greater survival from the initial phase of life-threatening illness (1–3). This achievement, however, has revealed a concomitant issue: an increasing number of patients who survive the immediate crisis subsequently enter a state of prolonged ICU dependency, primarily driven by unresolved organ dysfunction (4–7). This phenomenon, occurring in approximately 7.6% of ICU patients, is termed Chronic Critical Illness (CCI) (8). Mortality in CCI remains substantial high, with in-hospital and one-year mortality rates of 27 and 45%, respectively (9). Furthermore, only 24% of these patients are discharged directly home, while three out of four require transfer to long-term care or rehabilitation facilities (9). Despite its clinical significance, a universally accepted definition of CCI is still lacking (9–12).

A central challenge in CCI research is the unresolved pathophysiology driving the transition from acute to chronic critical illness. Two primary models have been proposed: the traditional two-phase model and a more recent three-stage model (13). The ongoing debate reflects a fundamental gap in evidence, stemming from an incomplete understanding of the pathological processes underlying critical illness chronicity (14, 15).

A leading pathophysiological candidate is the triad of persistent inflammation, immunosuppression, and catabolism, often referred to as PICS (Persistent Inflammation, Immunosuppression, and Catabolism Syndrome) (16). However, two key ambiguities surround this concept: the lack of a standardized definition and an unclear understanding of its relationship with CCI. The classic definition of PICS incorporates the triad alongside a mandatory time criterion – prolonged ICU stay (16). An emerging perspective advocates for a definition focused solely on the pathophysiology independent of a specific time interval (the ICS triad), which may offer greater clinical utility (13). The relationship between ICS and CCI remains to be fully elucidated, with several possibilities: ICS as the core pathophysiology of CCI (13, 16), ICS as a consequence of CCI (17), or ICS as a distinct endotype within the CCI spectrum (5). Crucially, the independent prognostic value of the ICS triad on patient outcomes is poorly characterized.

Therefore, the objective of this study was to investigate the impact of developing the inflammation, immunosuppression, and catabolism triad on hospitalization outcomes in critically ill patients.

## 2 Materials and methods

### 2.1 Data sources

This real-world data analysis utilized electronic health records (EHRs) from the Federal Research and Clinical Center of Intensive Care Medicine and Rehabilitology (Russian Intensive Care Dataset, RICD (18, 19)). The data covered all patients admitted to the intensive care units (ICUs) from December 2017 to July 2023. The local Ethics Committee confirmed that no formal approval was required for this study due to the de-identified nature of the data.

### 2.2 Selection criteria

We included all adult ICU patients who could be evaluated for the presence of ICS during their hospitalization. Daily ICS presence was determined based on three criteria: C-reactive protein (CRP) levels above 20 mg/L, albumin levels below 30 g/L, and lymphocyte counts under 0.8*10^9/L (20). Exclusion criteria were: (1) ICU stay of less than 24 h, (2) readmission to the ICU, and (3) absence of ICD-10 diagnostic information.

### 2.3 Data extraction

The data were extracted using SQLite version 3.45.2 and DBeaver software application. The parameters analyzed included the following: (1) general patient information: sex, age, body mass index (BMI), and whether the patient was transferred from another hospital; (2) sequential organ failure assessment (SOFA), Full Outline of UnResponsiveness (FOUR), Glasgow coma scale (GCS), and comorbidity; (3) laboratory data: white blood cell (WBC) count, neutrophil and lymphocyte counts, neutrophil-to-lymphocyte ratio (NLR), platelet count, international normalized ratio (INR), albumin, total protein, lactate, ALT, AST, creatinine, and CRP; and (4) hospitalization outcomes: hospital mortality, length of hospital stay, need for mechanical ventilation (MV), use of vasoactive/inotrope drugs, multiple organ failure, unfavorable outcome (patients who died or were transferred to another ICU), and discharge location. For patients with multiple ICS-related measurements on the same day, the highest CRP and the lowest albumin and lymphocyte counts were analyzed. For patients with ICS, the day of ICS onset was determined; for others, the last day on which ICS assessment was possible was evaluated. The timing and frequency of laboratory measurements were not protocol-driven but were performed at the attending physician’s discretion according to the patient’s clinical condition, severity, and disease dynamics, reflecting real-world clinical practice.

### 2.4 Outcomes

The primary endpoint was the risk of developing ICS among ICU patients. Secondary endpoints included all-cause hospital mortality, need for MV and vasoactive/inotrope drugs usage, and the duration of hospitalization. The duration of ICU and hospital stay were defined as the total length of stay in the ICU and hospital, respectively.

### 2.5 Statistical analysis

Data distribution was assessed using the Shapiro–Wilk test. Continuous variables were reported as medians (Me) and interquartile ranges (IQRs), while categorical variables were expressed as frequencies and percentages. The chi-square test and Fisher’s exact test (with Fisher–Freeman–Halton Exact extension where applicable) were used for comparing categorical variables. The Kruskal-Wallis test and Mann–Whitney U test (Wilcoxon rank-sum test) were applied for continuous variables.

Survival analysis was performed using Kaplan–Meier survival and failure curves, with cumulative risk functions estimated via the Nelson–Aalen estimator. Differences in survival between groups were evaluated using the log-rank test. Multivariable Cox regression analyses were conducted to identify independent predictors of ICS outcomes, with backward feature selection (Wald) revealing adjusted hazard ratios (HRs) and 95% confidence intervals (CIs). The proportional hazards assumption for Cox regression models was assessed using Schoenfeld residuals.

Missing data were not imputed. Statistical significance was set at *p* < 0.05 (two-sided). All the statistical calculations were performed using IBM SPSS Statistics v.29.0 and Stata v.18.0.

## 3 Results

### 3.1 Patient characteristics

**Figure 1:**
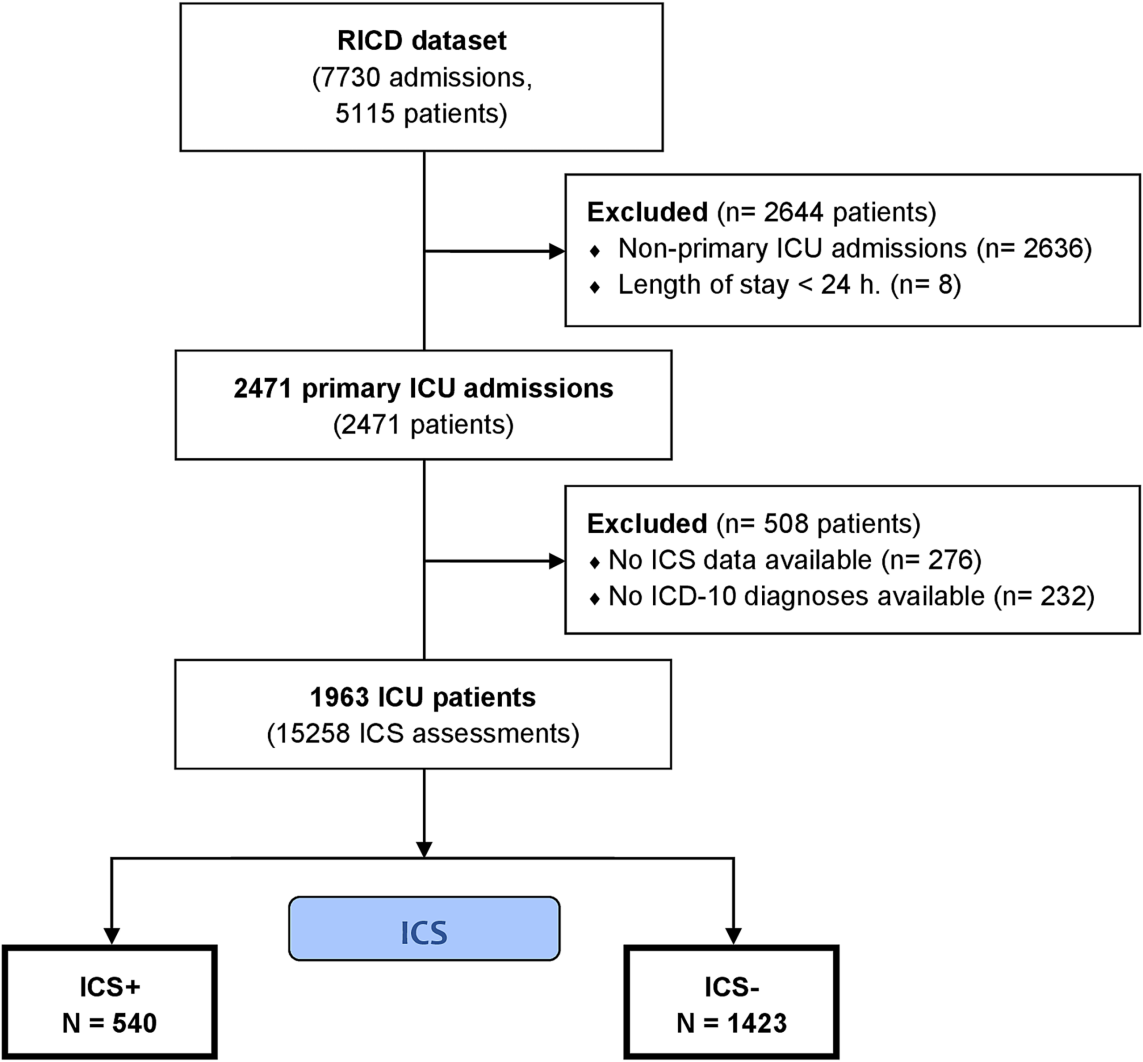
Flowchart of patient selection in the study.

A flowchart of patient selection for the study is presented in Figure 1. After applying the exclusion criteria, a total of 3,152 patients were excluded, resulting in an analysis cohort of 1963 patients (55% male). Among these, 540 patients (27.5%) developed ICS. The median age of the cohort was 62 years (IQR 49–73). Multiple organ failure developed in 1250 patients (64%), mechanical ventilation was required for 940 patients (48%), and vasoactive/inotrope drugs were administered to 303 patients (15%). Hospital mortality rate was 12%, with a median hospital stay of 34 days (IQR 22–50). More than half of the patients were transferred to another ICU, while the remainder were discharged for further treatment or rehabilitation (Table 1). The STROBE checklist for observational studies is provided in Supplementary Table 1.

**Table 1:** Initial parameters, disease characteristics, and outcomes.

Parameters	ICS, *N* = 540	No ICS, *N* = 1,423	*p*-value
Sex, male	280, 52%	808, 57%	0.05^1^
Age, years	69.0 (57.0–79.0)	60.0 (45.0–70.0)	**<0.001** ^**2**^
BMI, kg/m^2^	*N* = 495 (91.67%), 24.8 (21.5–29.3)	*N* = 1,282 (90.09%), 25.2 (21.8–28.7)	0.8^2^
Transfer from other hospital	518, 96%	1,374, 97%	0.5^3^
SOFA at admission, score	*N* = 389 (72.04%), 4.0 (2.0–5.0)	*N* = 981 (68.94%), 2.0 (1.0–4.0)	**<0.001** ^**2**^
FOUR at admission, score	*N* = 408 (75.56%), 13.0 (10.0–16.0)	*N* = 1,030 (72.38%), 16.0 (13.0–16.0)	**<0.001** ^**2**^
GCS at admission, score	*N* = 410 (75.93%), 11.0 (8.0–14.0)	*N* = 1,060 (74.49%), 14.0 (10.0–15.0)	**<0.001** ^**2**^
Comorbidity			
Ischemic stroke	289, 53%	652, 46%	**0.002** ^**1**^
Hemorrhagic stroke	81, 15%	236, 17%	0.4^1^
Traumatic brain injury	63, 12%	221, 16%	**0.036** ^**1**^
Anemia	27, 5.0%	55, 3.9%	0.3^3^
Type 2 diabetes	24, 4.4%	81, 5.7%	0.3^3^
Cardiovascular disease	10, 1.9%	28, 2.0%	0.9^3^
Chronic kidney disease	12, 2.2%	17, 1.2%	0.097^3^
Chronic obstructive pulmonary disease	6, 1.1%	8, 0.6%	0.2^3^
Pneumonia	248, 46%	459, 32%	**<0.001** ^**1**^
Myocardial infarction	1, 0.2%	8, 0.6%	0.5^3^
Coronary artery disease	134, 25%	254, 18%	**<0.001** ^**1**^
Atrial fibrillation	22, 4.1%	47, 3.3%	0.4^3^
Arterial hypertension	345, 64%	927, 65%	0.6^1^
Coagulopathy	4, 0.7%	3, 0.2%	0.1^3^
Inflammatory disorders of the CNS	7, 1.3%	15, 1.1%	0.6^3^
Polyneuropathy	4, 0.7%	8, 0.6%	0.7^3^
Heart failure	147, 27%	262, 18%	**<0.001** ^**1**^
Sepsis	27, 5.0%	13, 0.9%	**<0.001** ^**3**^
Valvular heart disease	8, 1.5%	8, 0.6%	0.052^3^
Mental and cognitive disorders	106, 20%	336, 24%	0.07^1^
Polytrauma	16, 3%	45, 3%	0.9^3^
Brain disorders	460, 85%	1,191, 84%	0.5^1^
Malignant tumor	18, 3.3%	36, 2.5%	0.4^3^
Laboratory parameters at admission			
WBC, 10^9/L	9.1 (6.9–11.9)	8.5 (6.6–11.2)	**0.008** ^**2**^
Lymphocyte count, 10^9/L	0.8 (0.6–1.2)	1.4 (1.1–1.9)	**<0.001** ^**2**^
Neutrophil count, 10^9/L	7.2 (5.1–10.2)	5.9 (4.2–8.5)	**<0.001** ^**2**^
NLR	8.6 (5.0–13.8)	4.0 (2.6–6.4)	**<0.001** ^**2**^
Platelets, 10^9/L	248.0 (183.0–335.0)	290.0 (226.0–370.8)	**<0.001** ^**2**^
INR	1.2 (1.1–1.4)	1.1 (1.1–1.3)	**<0.001** ^**2**^
Albumin, g/L	*N* = 470 (87.04%), 28.5 (25.2–32.5)	*N* = 1,219 (85.66%), 33.5 (29.3–37.4)	**<0.001** ^**2**^
Total protein, g/L	*N* = 515 (95.37%), 57.6 (52.4–63.4)	*N* = 1,360 (95.57%), 63.3 (58.0–68.1)	**<0.001** ^**2**^
Lactate, mmol/L	*N* = 279 (51.67%), 1.4 (1.0–2.0)	*N* = 602 (42.30%), 1.4 (1.0–1.9)	0.6^2^
ALT, U/L	*N* = 513 (95.00%), 26.1 (15.8–45.3)	*N* = 1,362 (95.71%), 28.7 (17.7–50.8)	**0.01** ^**2**^
AST, U/L	*N* = 513 (95.00%), 30.0 (20.7–45.5)	*N* = 1,359 (95.50%), 29.3 (20.7–43.5)	0.3^2^
Creatinine, μmol/L	*N* = 515 (95.37%), 78.8 (59.0–109.9)	*N* = 1,366 (95.99%), 75.7 (60.8–93.5)	**0.026** ^**2**^
CRP, mg/L	*N* = 505 (93.52%), 58.0 (33.2–124.6)	*N* = 1,297 (91.15%), 32.7 (9.9–68.9)	**<0.001** ^**2**^
Outcomes			
Hospital mortality	165, 31%	70, 4.9%	**<0.001** ^3^
Hospital length of stay, days			
All patients	34.0 (22.0–61.0)	33.0 (23.0–48.0)	0.16^2^
Only survived patients	*N* = 375 (69.44%), 40.0 (23.0–66.0)	*N* = 1,353 (95.08%), 34.0 (23.0–49.0)	**<0.001** ^**2**^
Need for MV	362, 67%	578, 41%	**<0.001** ^1^
Use of vasoactive/inotrope drugs	180, 33%	123, 8.6%	**<0.001** ^1^
Multiple organ failure	445, 82%	805, 56%	**<0.001** ^1^
Unfavorable outcome	417, 77%	572, 40%	**<0.001** ^1^
Discharge location			
ICU	416, 77%	569, 40%	**<0.001** ^**3**^
Palliative psych. Ward	84, 16%	433, 30%	
Neurorehabilitation	40, 7.4%	421, 30%	

^1^Chi-square test with continuity correction; ^2^Mann–Whitney U test; ^3^Fisher’s exact test.

ICS, inflammation, catabolism, and immunosuppression; ICU, intensive care unit; BMI, body mass index; SOFA, Sequential Organ Failure Assessment; FOUR, Full Outline of UnResponsiveness; GCS, Glasgow coma scale; MV, mechanical ventilation; WBC, white blood cell count; NLR, neutrophil-to-lymphocyte ratio; INR, International Normalized Ratio; CRP, C-reactive protein; CNS, central nervous system. *p*-values less than 0.05 are highlighted in bold.

### 3.2 Cumulative risk of ICS development

**Figure 2:**
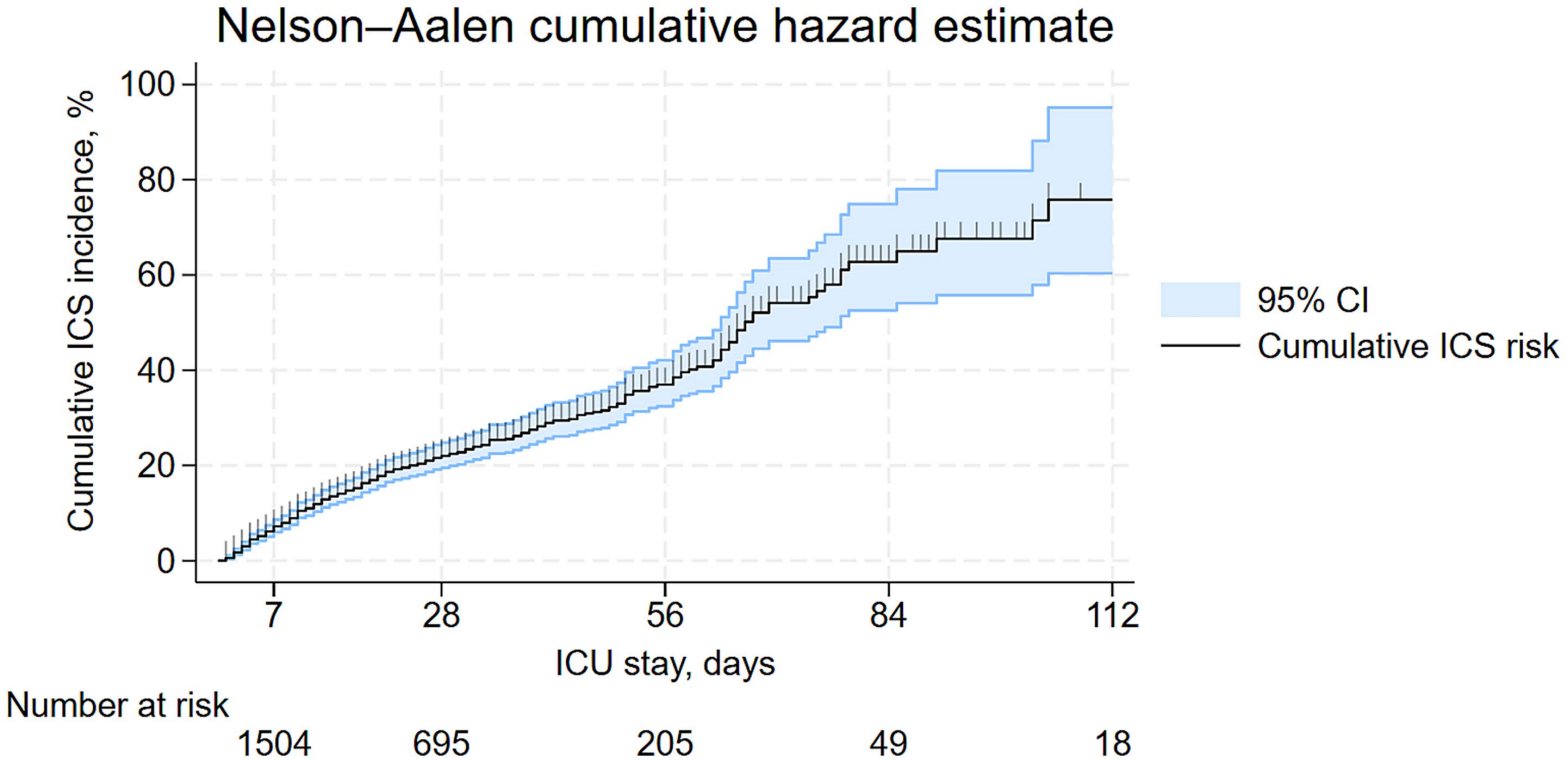
Cumulative risk of ICS development in ICU.

The proportion of patients with ICS in the study was 27.5%. The cumulative risk of developing ICS was 6.3% (95% CI 5.6–8.0) by day 7 of the ICU stay, 21.3% (95% CI 18.7–24.6) by day 28, and 76.1% (95% CI 60.4–95.3) by 16 weeks of ICU stay (Figure 2).

### 3.3 ICS risk factors

Patients who developed ICS were significantly older, with a median age of 69 years compared to 60 years in those without ICS (*p* < 0.001). At admission, these patients had higher SOFA scores (median 4.0 vs. 2.0, *p* < 0.001) and lower scores on the FOUR (median 13.0 vs. 16.0, *p* < 0.001) and GCS (median 11.0 vs. 14.0, *p* < 0.001) scales.

Comorbidity profiles differed between the groups: ICS patients had a higher prevalence of pneumonia (46% vs. 32%, *p* < 0.001), heart failure (27% vs. 18%, *p* < 0.001), sepsis (5.0% vs. 0.9%, *p* < 0.001), and coronary artery disease (25% vs. 18%, *p* < 0.001). Conversely, ischemic stroke (53% vs. 46%, *p* = 0.002) and traumatic brain injury (12% vs. 16%, *p* = 0.036) were less common in ICS patients.

Patients with ICS had elevated WBC counts (median 9.1 vs. 8.5 × 10^9/L, *p* = 0.008), neutrophil counts (median 7.2 vs. 5.9 × 10^9/L, *p* < 0.001), NLR (median 8.6 vs. 4.0, *p* < 0.001), INR (median 1.2 vs. 1.1, *p* < 0.001), and creatinine levels (median 78.8 vs. 75.7 μmol/L, *p* = 0.026). Conversely, ICS patients had lower platelet counts (median 248.0 vs. 290.0 × 10^9/L, *p* < 0.001), albumin levels (median 28.5 vs. 33.5 g/L, *p* < 0.001), and total protein levels (median 57.6 vs. 63.3 g/L, *p* < 0.001). Notably, CRP levels at admission were markedly higher in ICS patients (median 58.0 vs. 32.7 mg/L, *p* < 0.001).

### 3.4 ICS outcomes

**Figure 3:**
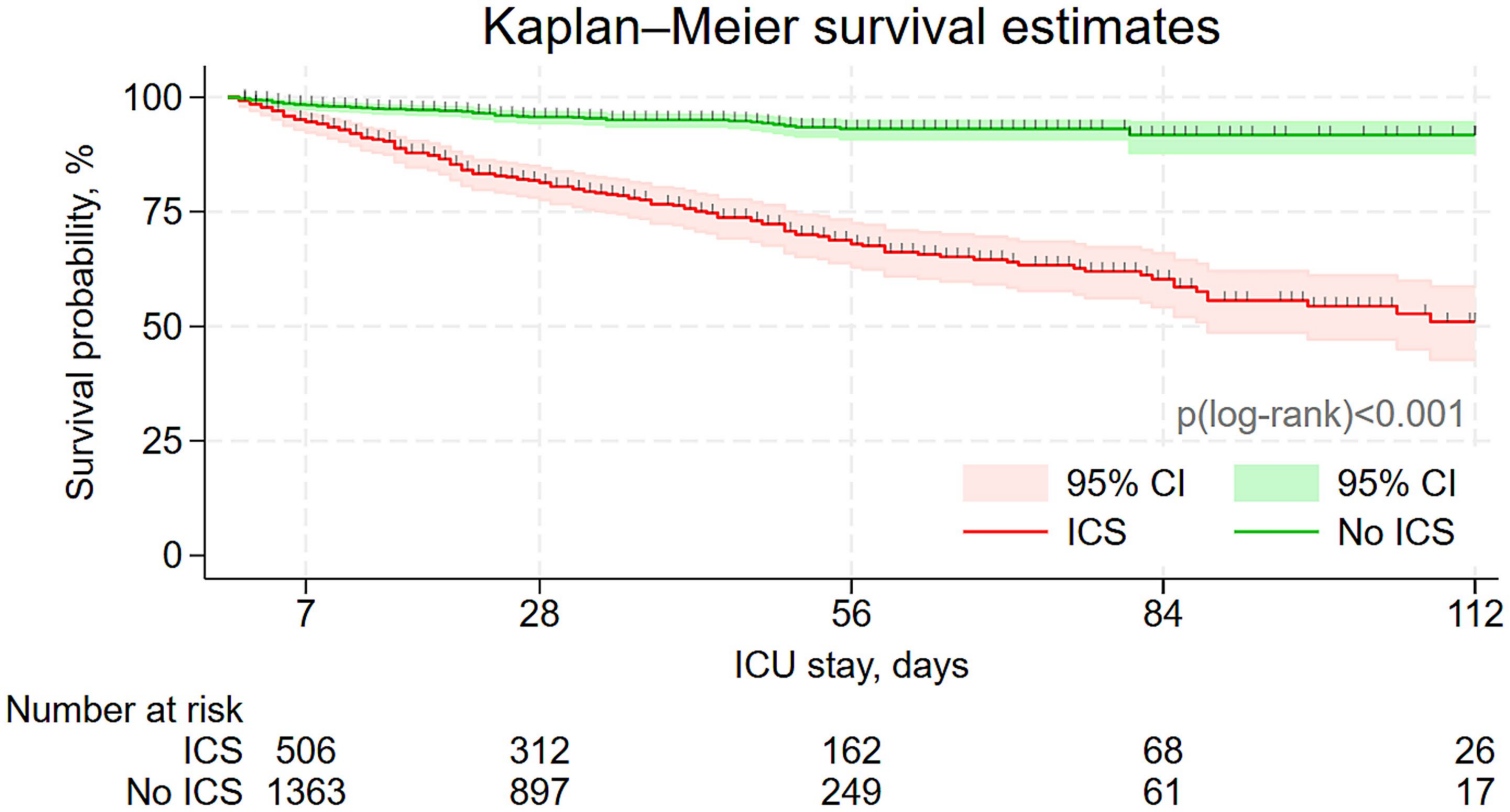
Kaplan–Meier survival curves showing the impact of the ICS on survival in ICU patients.

**Figure 4:**
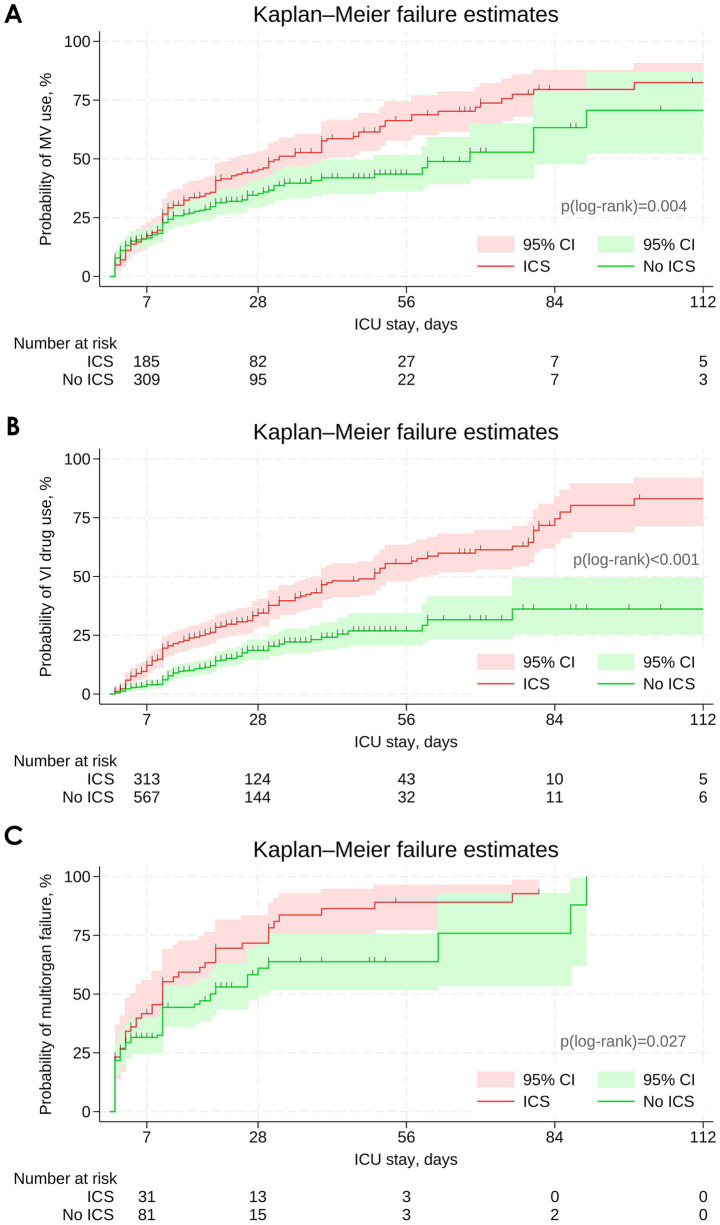
Kaplan–Meier failure curves showing the impact of the ICS on the use of mechanical ventilation **(A)**, use of vasoactive/inotrope drugs **(B)**, and occurrence of multiple organ failure **(C)**.

Hospital mortality was significantly higher in ICS patients (relative risk 6.21 [95% CI 4.78; 8.07], 31% vs. 4.9%, *p* < 0.001). Kaplan–Meier survival analysis demonstrated poorer survival for ICS patients, with a mean ICU survival time of 120 days (95% CI 106–135) compared to 200 days (95% CI 180–220, *p* < 0.001) for non-ICS patients (Figure 3). The need for mechanical ventilation was increased among ICS patients (67% vs. 41%, *p* < 0.001), with a relative risk of 1.7 (95% CI 1.5–1.8). Additionally, the use of vasoactive/inotrope drugs was more frequent in the ICS group (33% vs. 8.6%, *p* < 0.001), with a relative risk of 3.9 (95% CI 3.1–4.7) (Figure 4).

Patients with ICS also had longer hospital stays among survivors (median 40 days vs. 34 days, *p* < 0.001). They exhibited higher rates of multiple organ failure (82% vs. 56%, *p* < 0.001) (Figure 4) and unfavorable outcomes (77% vs. 40%, *p* < 0.001).

In the univariate analysis, individual baseline ICS components demonstrated significant associations with hospital mortality: CRP levels above 20 mg/L (HR 2.81, 95% CI 1.90–4.16, *p* < 0.001), albumin levels below 30 g/L (HR 2.20, 95% CI 1.67–2.91, *p* < 0.001), and lymphocyte counts under 0.8 × 10^9/L (HR 3.39, 95% CI 2.59–4.45, *p* < 0.001).

Multivariable Cox regression analysis identified ICS as an independent predictor of hospital mortality, with an adjusted hazard ratio of 2.16 (95% CI 1.28–3.65, *p* = 0.004). Other significant predictors included SOFA score at admission (HR 1.22, 95% CI 1.13–1.33, *p* < 0.001), NLR at admission (HR 1.019, 95% CI 1.002–1.037, *p* = 0.029), lactate levels at admission (HR 1.18, 95% CI 1.03–1.35, *p* = 0.020), chronic kidney disease (HR 3.03, 95% CI 1.13–8.12, *p* = 0.028), coronary artery disease (HR 2.26, 95% CI 1.31–3.90, *p* = 0.003), and arterial hypertension (HR 1.99, 95% CI 1.16–3.43, *p* = 0.013) (Table 2). ICS was also an independent predictor of use of vasoactive/inotrope drugs (HR 1.85, 95% CI 1.29–2.64, *p* < 0.001). Moreover, ICS was the only independent risk factor for multiple organ failure (HR 1.25, 95% CI 1.05–1.47, *p* = 0.011), Table 2.

**Table 2:** Cox regression analysis.

Parameters in initial model	Significant parameters in final model	Multivariable analysis adj. HR (95% CI)	*p* value
Hospital mortality (step 26)			
Age, sex, BMI, ICS, SOFA, total protein, NLR, INR, ALT, AST, lactate, all comorbidities	ICS presence	2.16 (1.28–3.65)	0.004
	SOFA at admission	1.22 (1.13–1.33)	<0.001
	NLR at admission	1.019 (1.002–1.037)	0.029
	Lactate at admission	1.18 (1.03–1.35)	0.020
	Chronic kidney disease	3.03 (1.13–8.12)	0.028
	Coronary artery disease	2.26 (1.31–3.90)	0.003
	Arterial hypertension	1.99 (1.16–3.43)	0.013
Need for mechanical ventilation (step 25)			
Age, sex, BMI, ICS, total protein, NLR, INR, ALT, AST, lactate, all comorbidities	BMI	1.02 (1.003–1.04)	0.021
	Total protein at admission	0.97 (0.96–0.99)	<0.001
	NLR at admission	1.01 (1.002–1.02)	0.021
	Pneumonia	1.65 (1.33–2.04)	<0.001
	Inflammatory disorders of the CNS	2.52 (1.12–5.71)	0.026
Use of vasoactive/inotrope drugs (step 29)			
Age, sex, BMI, ICS, SOFA, total protein, NLR, INR, ALT, AST, lactate, all comorbidities (excl. Heart failure)	ICS presence	1.85 (1.29–2.64)	<0.001
	SOFA at admission	1.30 (1.23–1.38)	<0.001
	Atrial fibrillation	2.40 (1.13–4.40)	0.004
	Sepsis	1.89 (1.02–3.49)	0.043
Multiple organ failure (step 31)			
Age, sex, BMI, ICS, total protein, NLR, INR, ALT, AST, lactate, all comorbidities	ICS presence	1.25 (1.05–1.47)	0.011

ICS, inflammation, catabolism, and immunosuppression; ICU, intensive care unit; BMI, body mass index; SOFA, Sequential Organ Failure Assessment; FOUR, Full Outline of UnResponsiveness; GCS, Glasgow coma scale; NLR, neutrophil-to-lymphocyte ratio; INR, International Normalized Ratio; HR, hazard ratio; CI, confidence interval.

## 4 Discussion

### 4.1 Key findings

Results from a real-world data analysis of 1,963 patients revealed an ICS incidence of 27.5%. The cumulative risk of developing ICS was 21.3% by day 28 and 76.1% by week 16 of ICU stay. Hospital mortality was significantly higher in patients with ICS (31% vs. 4.9%, *p* < 0.001), as was the frequency of mechanical ventilation (67% vs. 41%, *p* < 0.001) and the use of vasoactive/inotropic support (33% vs. 8.6%, *p* < 0.001). The ICS triad was identified as an independent risk factor for both hospital mortality (HR = 2.16; 95% CI 1.28–3.65, *p* = 0.004) and the need for vasoactive/inotropic drugs (HR 1.85, 95% CI 1.29–2.64, *p* < 0.001). Furthermore, ICS was the sole independent risk factor for the development of multiple organ failure (HR 1.25, 95% CI 1.05–1.47, *p* = 0.011).

### 4.2 Relationship with previous studies

The lack of standardized diagnostic criteria for ICS complicates direct comparisons of our findings with other studies. Nonetheless, several investigations focusing on ICS outcomes have reported similar results.

For instance, Dong Hu et al. demonstrated significantly higher mortality in patients with the ICS triad compared to those with prolonged ICU stays without ICS (28.3% vs. 7.1%, *p* = 0.002), a finding which closely aligns with our results (21). Their study also showed a greater need for mechanical ventilation (79.2% vs. 61.4%, *p* = 0.048), further corroborating our data. In addition, the study by Uyar S. et al. also demonstrated the prognostic significance of the triad components with respect to 28-day mortality in septic patients (22).

Similarly, a retrospective study by Nakamura K et al. confirmed a higher mortality risk in ICS patients (13.9% vs. 7.0%, *p* = 0.012) (23). The notable difference in absolute mortality rates between their study and ours may be primarily attributed to an immortal time bias in the Nakamura et al. cohort, which exclusively enrolled patients hospitalized for more than 14 days, whereas our study included all patients who survived the first 24 h of admission.

Consistent with our findings, a recent study by Hui et al. (2024) in a large cohort of septic patients (*n* = 1733) demonstrated that the presence of PICS was associated with significantly higher mortality (38.08% vs. 19.48%, *p* < 0.001) and longer ICU stays compared to non-PICS patients (24). In that cohort, PICS patients also required mechanical ventilation, vasopressor support, and renal replacement therapy more frequently, and had a higher prevalence of multidrug-resistant infections – which are highly consistent with the patterns observed in our cohort. These results reinforce the notion that the clinical trajectory of patients with persistent inflammation, immunosuppression, and catabolism is marked by a substantially worse prognosis, regardless of the specific predictive approach used.

Similarly, a single-center observational study by Zhong et al. (25) in surgical sepsis patients reported that those who developed PICS had significantly higher ICU mortality (32.4% vs. 12.4% after propensity score matching, *p* = 0.046) and 90-day mortality (32.4% vs. 9.1%, *p* = 0.006) compared to non-PICS patients. The PICS group also exhibited a markedly higher rate of ICU-acquired infections (44.1% vs. 12.7%, *p* < 0.001) and longer ICU stays (29 vs. 11 days, *p* < 0.001). These findings are remarkably similar to ours, as we observed comparable elevations in mortality, infectious complications, and resource utilization among patients with the PICS triad. The consistency across different cohorts – surgical sepsis in the Zhong study and our ICU population – underscores the robust association between PICS and adverse clinical outcomes, irrespective of the underlying patient mix.

Our results are also consistent with our previous work (13) utilizing the eICU Collaborative Research Database (eICU-CRD) (26). In the study by Likhvantsev VV et al., in-hospital mortality was significantly higher in the ICS group (18.3% vs. 4.9%, *p* < 0.001). The requirement for vasoactive drugs in that study was twice as high among ICS patients compared to non-ICS patients with prolonged ICU stays (27% vs. 14%, *p* = 0.008), which parallels the trend observed in our cohort (33% vs. 8.6%). Furthermore, the cumulative risk curves for ICS development were remarkably synchronous between the two studies.

### 4.3 Significance of study findings

The clinical relevance of our findings is underscored by several key observations.

#### 4.3.1 The observed association of ICS with adverse outcomes and its potential relevance to the late multiple organ failure hypothesis

This study provides evidence that the development of the inflammation, immunosuppression, and catabolism syndrome (ICS) worsens hospitalization outcomes. A key finding is that the adverse prognostic effect of ICS is not contingent upon a predefined ICU length-of-stay threshold. The mere presence of the triad significantly elevates the risk of hospital mortality, mechanical ventilation dependence, and the need for vasopressor support. Moreover, among all variables analyzed, ICS was the only independent risk factor for the onset of late multiple organ failure. These results offer preliminary evidence in support of the pathophysiological model of late multiple organ failure proposed by Lori F. Gentile et al. in 2012 (16). Certainly, the components of the ICS triad (inflammation, immunosuppression, catabolism) are biologically interrelated with the pathogenesis of multiple organ failure, and some conceptual overlap is therefore inevitable. Nevertheless, ICS remained an independent risk factor for MOF in the multivariable analysis, suggesting additional prognostic value of the combined triad.

#### 4.3.2 A distinct cohort: prolonged ICU stay without ICS

A significant and insightful finding is the identification of a distinct cohort of patients who, despite prolonged ICU stays of several months, never developed ICS. This group exhibited significantly better outcomes than ICS patients. The existence of this cohort prompts a key question: can all three triad components be present without fulfilling the syndromic diagnostic threshold? Our data lend support to the concept of temporal dissociation or asynchrony among the triad components. Laboratory analysis revealed that some non-ICS patients presented with baseline inflammation and hypoalbuminemia upon admission. This suggests that individual components may reach pathological levels at different times. Isolated disturbances in one or two pathways may be physiologically compensated. However, the simultaneous and persistent dysregulation of all three axes appears to overwhelm homeostatic mechanisms, creating a self-perpetuating pathological cycle that drives increased disease severity and poor prognosis.

#### 4.3.3 Clarifying the CCI-ICS relationship

The pathophysiological link between chronic critical illness and the inflammation, immunosuppression, and catabolism syndrome remains debated, with ICS proposed as a cause, consequence, or endotype of CCI within the two-phase model (27), or as the central driver of chronicity in the three-stage model (13). The results of the present study clarify the relationship between these concepts. The cumulative risk curves for ICS development, derived from both the study by Likhvantsev VV et al. (13) and this analysis, indicate that the proportion of patients developing the triad increases with ICU length of stay, suggesting our understanding is limited primarily by the duration of observation. It is highly probable that, given sufficient hospitalization time, nearly all patients would eventually develop ICS. Moreover, in a subset of patients, the triad is diagnosed early in the clinical course (within 7 days of the acute event). The early onset of ICS makes it unlikely that it is solely a late consequence of established CCI, and raises the possibility that it may represent an early contributing factor in the chronic critical illness trajectory. Overall, these findings are consistent with the view that the ICS triad may represent a contributing factor to CCI, and it does not appear to be exclusively an outcome or a separate endotype based on our data.

#### 4.3.4 Refining the model of critical illness chronicity

Our findings regarding the timing of ICS onset and its relationship with CCI necessitate a reevaluation of existing models of critical illness chronicity. The following interpretation of the Three-steps model is offered as a theoretical framework to contextualize our observations, and should not be taken as a validated pathophysiological construct.

Within the traditional two-phase model, positioning ICS as the core pathophysiology of CCI appears logical. This framework would then classify all patients without the triad as being in an acute critical illness state. However, this classification becomes problematic, as it seems counterintuitive to label patients who have remained in the ICU for months without developing ICS as still being in the acute phase of their initial illness.

The three-stage model, which posits ICS as the driver of the transition from acute to prolonged and, ultimately, to chronic critical illness, offers a more nuanced view. Yet, faced with our evidence, it encounters a similar conceptual challenge: if the transition between stages is defined by the emergence of ICS, then patients with protracted ICU stays who never develop the triad must also be categorized as remaining in the acute stage. While the two-phase model appears inadequate and difficult to reconcile with these new data, the three-stage model can be productively revised.

**Figure 5:**
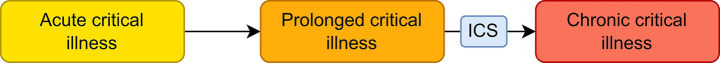
Hypothetical three-step model of the transition to chronic critical illness.

We propose a revised interpretation: the development of ICS signifies the establishment of a self-perpetuating pathological cycle that defines CCI. In essence, the simultaneous dysregulation of all three axes confirms the transition from a prolonged to a chronic critical state. This refinement, however, leaves the precise transition point from acute to prolonged illness indeterminate. Our data suggest that the duration of this “prolonged critical state” can vary widely (from days to months) during which compensatory mechanisms may prevent the onset of the full ICS triad (Figure 5).

Importantly, Kaplan–Meier analysis from our study indicates that this prolonged state represents a critical therapeutic window where interventions may be most effective in preventing disease progression. In contrast, once the chronic phase (marked by established ICS) is entered, patient prognosis deteriorates significantly despite intensive care.

Based on these observations, one might hypothesize that future approaches to long-term ICU patients could benefit from greater attention to the pathophysiological processes occurring during the prolonged critical state, rather than focusing solely on treating established chronic critical illness after it develops.

### 4.4 Strengths and limitations

This study has several key strengths, including the use of a large, real-world cohort that enhances generalizability and the application of standardized diagnostic criteria for ICS, a notable advancement given the current lack of definitional consensus. The extended follow-up period allowed for a detailed analysis of the syndrome’s time-dependent nature, while rigorous multivariable adjustment confirmed ICS as an independent risk factor for major adverse outcomes. Furthermore, the strong concordance of our cumulative incidence curves with those from an external, multicenter database provides compelling support for the observed temporal dynamics.

The study’s limitations must also be acknowledged. Its single-center design inherently limits causal inference and may affect generalizability due to local practice patterns. While we adjusted for numerous confounders, residual confounding by unmeasured variables remains possible given the absence of data on genetic polymorphisms affecting inflammatory responses, detailed medication history (including corticosteroids and immunomodulators), and pre-ICU nutritional or functional status. Furthermore, our cohort included a predominance of neurological diagnoses (ischemic and hemorrhagic stroke, traumatic brain injury), which may limit the generalizability of our findings to other ICU populations such as surgical, trauma, septic, or cardiac intensive care settings. Moreover, the study is subject to immortal time bias, a common challenge in observational studies of time-dependent exposures. Because patients must survive long enough to develop the ICS triad, those who die or are discharged early are inherently excluded from the exposed group, whereas the unexposed group includes patients who may have died before ever meeting the ICS criteria. In our analysis, ICS was not modeled as a time-varying covariate, and we did not perform landmark analyses or delayed-entry methods. Consequently, the observed hazard ratios for ICS (e.g., HR 2.16 for mortality) may be overestimated, as part of the prognostic association could reflect survivorship bias rather than the direct biological effect of the syndrome itself. Specifically, patients who develop ICS are by definition those who have survived long enough to be assessed, which may artificially inflate the apparent protective effect of being ICS-free. This bias could affect not only the magnitude of the mortality hazard but also the estimates for other outcomes such as mechanical ventilation and vasopressor use. While we attempted to minimize this by using survival analysis with time-to-event data and adjusting for baseline covariates, these approaches do not fully eliminate the time-dependent nature of the exposure. Future studies should address this limitation by employing methods such as Cox regression with time-dependent covariates, landmark analysis at fixed time points (e.g., day 7 or day 14), or matching on duration of follow-up to better isolate the true effect of ICS independent of survivorship dynamics. Finally, our definition of ICS, based on specific biomarker thresholds, is pragmatic but may not capture all nuances of the syndrome.

### 4.5 Future studies and prospects

Three primary research trajectories emerge from our findings.

First, the proposed three-step model should be regarded as hypothesis-generating and requires prospective validation in independent cohorts. Future studies could help clarify whether ICS indeed represents a pathophysiological correlate of CCI and better characterizes the transition points between acute, prolonged, and chronic stages.

Second, our findings suggest that future studies should explore whether targeted interventions during the ‘prolonged critical state’ window could prevent progression to established CCI.

Third, future investigations should prospectively validate our findings using analytical strategies that explicitly account for immortal time bias, such as time-varying Cox models, landmark analyses, or propensity score matching with time-dependent covariates. These approaches would provide a more robust estimate of the independent contribution of the ICS triad to clinical outcomes.

## 5 Conclusion

In this real-world data analysis, the inflammation, immunosuppression, and catabolism syndrome was associated with hospital mortality and organ failure in ICU patients. These findings suggest that ICS may represent an important pathophysiological feature of chronic critical illness, though this interpretation remains hypothesis-generating and requires prospective validation. The identification of a potentially modifiable phase preceding the onset of ICS raises the possibility that earlier recognition and preventive strategies could be clinically relevant, but further interventional studies are needed before such approaches can be recommended.

## Data Availability

The original contributions presented in the study are included in the article/Supplementary material, further inquiries can be directed to the corresponding author.
