# Supplementary material for: The ICS triad in critical illness: a time-dependent pathological driver of mortality and organ dysfunction
Source: Front Med (Lausanne). 2026 Jul 14;13:1833525. doi: 10.3389/fmed.2026.1833525 (PMC13407986; doi:10.3389/fmed.2026.1833525)
Supplement: Supplementary file 1 [file Table_1.docx]

Supplementary Material

**Supplemental Table 1.** STROBE Statement – Checklist of items that should be included in reports of ***cohort studies***

|  | **Item No** | **Recommendation** | **Reported in** |
| --- | --- | --- | --- |
| **Title and abstract** | 1 | (*a*) Indicate the study’s design with a commonly used term in the title or the abstract | Title |
|  |  | (*b*) Provide in the abstract an informative and balanced summary of what was done and what was found | Abstract |
| **Introduction** | | | |
| Background/rationale | 2 | Explain the scientific background and rationale for the investigation being reported | Introduction |
| Objectives | 3 | State specific objectives, including any prespecified hypotheses | Introduction |
| **Methods** | | | |
| Study design | 4 | Present key elements of study design early in the paper | Data sources |
| Setting | 5 | Describe the setting, locations, and relevant dates, including periods of recruitment, exposure, follow-up, and data collection | Data sources |
| Participants | 6 | (*a*) Give the eligibility criteria, and the sources and methods of selection of participants. Describe methods of follow-up | Selection criteria |
|  |  | (*b*) For matched studies, give matching criteria and number of exposed and unexposed | NA |
| Variables | 7 | Clearly define all outcomes, exposures, predictors, potential confounders, and effect modifiers. Give diagnostic criteria, if applicable | Data extraction |
| Data sources/ measurement | 8* | For each variable of interest, give sources of data and details of methods of assessment (measurement). Describe comparability of assessment methods if there is more than one group | Data extraction |
| Bias | 9 | Describe any efforts to address potential sources of bias | Data extraction |
| Study size | 10 | Explain how the study size was arrived at | Data extraction |
| Quantitative variables | 11 | Explain how quantitative variables were handled in the analyses. If applicable, describe which groupings were chosen and why | Data extraction, Outcomes |
| Statistical methods | 12 | (*a*) Describe all statistical methods, including those used to control for confounding | Statistical analysis |
|  |  | (*b*) Describe any methods used to examine subgroups and interactions | Statistical analysis |
|  |  | (*c*) Explain how missing data were addressed | Statistical analysis |
|  |  | (*d*) If applicable, explain how loss to follow-up was addressed | Statistical analysis |
|  |  | (*e*) Describe any sensitivity analyses | Statistical analysis |
| **Results** | | |  |
| Participants | 13* | (a) Report numbers of individuals at each stage of study—eg numbers potentially eligible, examined for eligibility, confirmed eligible, included in the study, completing follow-up, and analysed | Patient characteristics |
|  |  | (b) Give reasons for non-participation at each stage | Patient characteristics |
|  |  | (c) Consider use of a flow diagram | Patient characteristics, Figure 1 |
| Descriptive data | 14* | (a) Give characteristics of study participants (eg demographic, clinical, social) and information on exposures and potential confounders | Patient characteristics |
|  |  | (b) Indicate number of participants with missing data for each variable of interest | Patient characteristics |
|  |  | (c) Summarise follow-up time (eg, average and total amount) | Patient characteristics |
| Outcome data | 15* | Report numbers of outcome events or summary measures over time | ICS outcomes |
| Main results | 16 | (*a*) Give unadjusted estimates and, if applicable, confounder-adjusted estimates and their precision (eg, 95% confidence interval). Make clear which confounders were adjusted for and why they were included | Cumulative risk of ICS development, ICS risk factors, ICS outcomes |
|  |  | (*b*) Report category boundaries when continuous variables were categorized | NA |
|  |  | (*c*) If relevant, consider translating estimates of relative risk into absolute risk for a meaningful time period | NA |
| Other analyses | 17 | Report other analyses done—eg analyses of subgroups and interactions, and sensitivity analyses | Cumulative risk of ICS development, ICS risk factors, ICS outcomes |
| **Discussion** | | | |
| Key results | 18 | Summarise key results with reference to study objectives | Key findings |
| Limitations | 19 | Discuss limitations of the study, taking into account sources of potential bias or imprecision. Discuss both direction and magnitude of any potential bias | Strengths and limitations |
| Interpretation | 20 | Give a cautious overall interpretation of results considering objectives, limitations, multiplicity of analyses, results from similar studies, and other relevant evidence | Significance of study findings |
| Generalisability | 21 | Discuss the generalisability (external validity) of the study results | Relationship with previous studies, Future studies and prospects |
| **Other information** | | | |
| Funding | 22 | Give the source of funding and the role of the funders for the present study and, if applicable, for the original study on which the present article is based | Funding information |

*Give information separately for exposed and unexposed groups.

**Note:** An Explanation and Elaboration article discusses each checklist item and gives methodological background and published examples of transparent reporting. The STROBE checklist is best used in conjunction with this article (freely available on the Web sites of PLoS Medicine at http://www.plosmedicine.org/, Annals of Internal Medicine at http://www.annals.org/, and Epidemiology at http://www.epidem.com/). Information on the STROBE Initiative is available at http://www.strobe-statement.org.
